# Blockage of AEP attenuates TBI-induced tau hyperphosphorylation and cognitive impairments in rats

**DOI:** 10.18632/aging.103841

**Published:** 2020-10-10

**Authors:** Yi Liu, Cuiping Guo, Yi Ding, Xiaobing Long, Wensheng Li, Dan Ke, Qun Wang, Rong Liu, Jian-Zhi Wang, Huaqiu Zhang, Xiaochuan Wang

**Affiliations:** 1 Department of Pathophysiology, Weifang Medical University, Weifang 261053, China; 2 Department of Pathophysiology, School of Basic Medicine, Key Laboratory of Education Ministry of China for Neurological Disorders, Tongji Medical College, Huazhong University of Science and Technology, Wuhan 430030, China; 3 Department of Neurosurgery, Tongji Hospital, Tongji Medical College, Huazhong, University of Science and Technology, Wuhan 430030, China; 4 Co-innovation Center of Neuroregeneration, Nantong University, Nantong 226001, JS, China

**Keywords:** traumatic brain injury (TBI), asparaginyl endopeptidase (AEP), AEP inhibitor/AENK, tau pathology, cognitive impairment

## Abstract

Traumatic brain injury (TBI) is regarded as a high-risk factor for Alzheimer's disease (AD). Asparaginyl endopeptidase (AEP), a lysosomal cysteine protease involved in AD pathogenesis, is normally activated under acidic conditions and also in TBI. However, both the molecular mechanism underlying AEP activation-mediated TBI-related AD pathologies, and the role of AEP as an AD therapeutic target, still remain unclear. Here, we report that TBI induces hippocampus dependent cognitive deficit and synaptic dysfunction, accompanied with AEP activation, I_2_^PP2A^ (inhibitor 2 of PP2A, also called SET) mis-translocation from neuronal nucleus to cytoplasm, an obvious increase in AEP interaction with SET, and tau hyperphosphorylation in hippocampus of rats. Oxygen-glucose deprivation (OGD), mimicking an acidic condition, also leads to AEP activation, SET mis-translocation, PP2A inhibition, tau hyperphosphorylation, and a decrease in synaptic proteins, all of which are abrogated by AEP inhibitor AENK in primary neurons. Interestingly, AENK restores SET back to the nucleus, mitigates tau pathologies, rescuing TBI-induced cognitive deficit in rats. These findings highlight a novel etiopathogenic mechanism of TBI-related AD, which is initiated by AEP activation, accumulating SET in cytoplasm, and favoring tau pathology and cognitive impairments. Lowering AEP activity by AEP inhibitor would be beneficial to AD patients with TBI.

## INTRODUCTION

As the current most common neurodegenerative disease, Alzheimer's disease (AD) is histopathologically characterized by the presence of two hallmark lesions: extracellular senile plaques consisting of β-amyloid and intracellular neurofibrillary tangles (NFTs) made up of the abnormally hyperphosphorylated tau [1–3]. Substantial evidence supports that the severity of clinical dementia is positively correlated with load and the spatial brain distribution of tangles in AD patients [4, 5], indicating that tau hyperphosphorylation is closely related with cognitive deficit in AD [6, 7]. Selective degeneration of inhibitory synapse with hyperexcitability constitutes the critical early pathophysiology of tauopathy [8]. Protein phosphatase-2A (PP2A), a major regulator of tau phosphorylation [9, 10], is obviously decreased in the AD brains compared to normal [11, 12], while the level of its inhibitor 2 (I_2_^PP2A^), also known as SET, is increased [13, 14]. SET is mostly localized in the nucleus [15, 16]. However, in the AD brains, SET is translocated from its primary nuclear location to the cytoplasm in neurons, and co-localizes with both PP2A and abnormally hyperphosphorylated tau in the neuronal cytoplasm [17, 18]. At present, the underlying molecular mechanism via which full length SET is delocalized and accumulates in the cytosol is not fully elucidated.

Asparaginyl endopeptidase (AEP) is a lysosomal cysteine protease, also known as δ-secretase [19–21]. In AD, AEP is highly active, causing the truncation of SET protein thereby promoting the nucleus to the cytoplasm translocation of SET, which indirectly leads to hyperphosphorylation of Tau [22–24]. Acidosis in brain tissue may also lead to tau hyperphosphorylation by promoting AEP autoactivation [25–27].

Traumatic brain injury (TBI) is a serious public health problem worldwide, leading to a substantial number of deaths and cases of permanent disability [28, 29]. TBI is a common phenomenon in the elderly, and its prognosis is further affected by the increase of age. At present, TBI cases among people over 65 have increased by 21% [30]. TBI has been identified as a major risk factor for chronic traumatic encephalopathy (CTE) and AD [31]. TBI arises from a bump, blow or jolt to the head or a penetrating head injury that disrupts the normal function of the brain [32–34]. Severe TBI might lead to unconsciousness or amnesia [35–37]. Increasing evidence shows that TBI patients are more prone to neurodegenerative diseases and memory defect, which is a major complication of TBI-related chronic brain injury [38, 39]. Interestingly, TBI was reported to induce an increase in lactic acid level in brain tissue [40, 41], which may help in AEP activation. We therefore speculated that AEP activation as a result of brain environment acidification following TBI might be the link between TBI and AD.

In the current study, behavioral testing showed that blocking or decreasing AEP activity attenuated TBI-induced learning and memory dysfunction in rats. On one hand, AEP inhibitor (AENK) restores SET back to its normal nucleus compartment, recovers tau phosphorylation in the hippocampus of TBI rats. On the other hand, AEP inhibitor is able to restore the reduction in the expression of synaptic proteins, the spines density and LTP damage. These data suggest that intervention with AEP inhibitor/AENK may contribute to the recovery of learning and memory function after TBI, and provide the basis for clinical intervention in neuropathological and behavioral complications of TBI.

## RESULTS

### Traumatic brain injury impaired cognitive functions in rats

**Figure 1:**
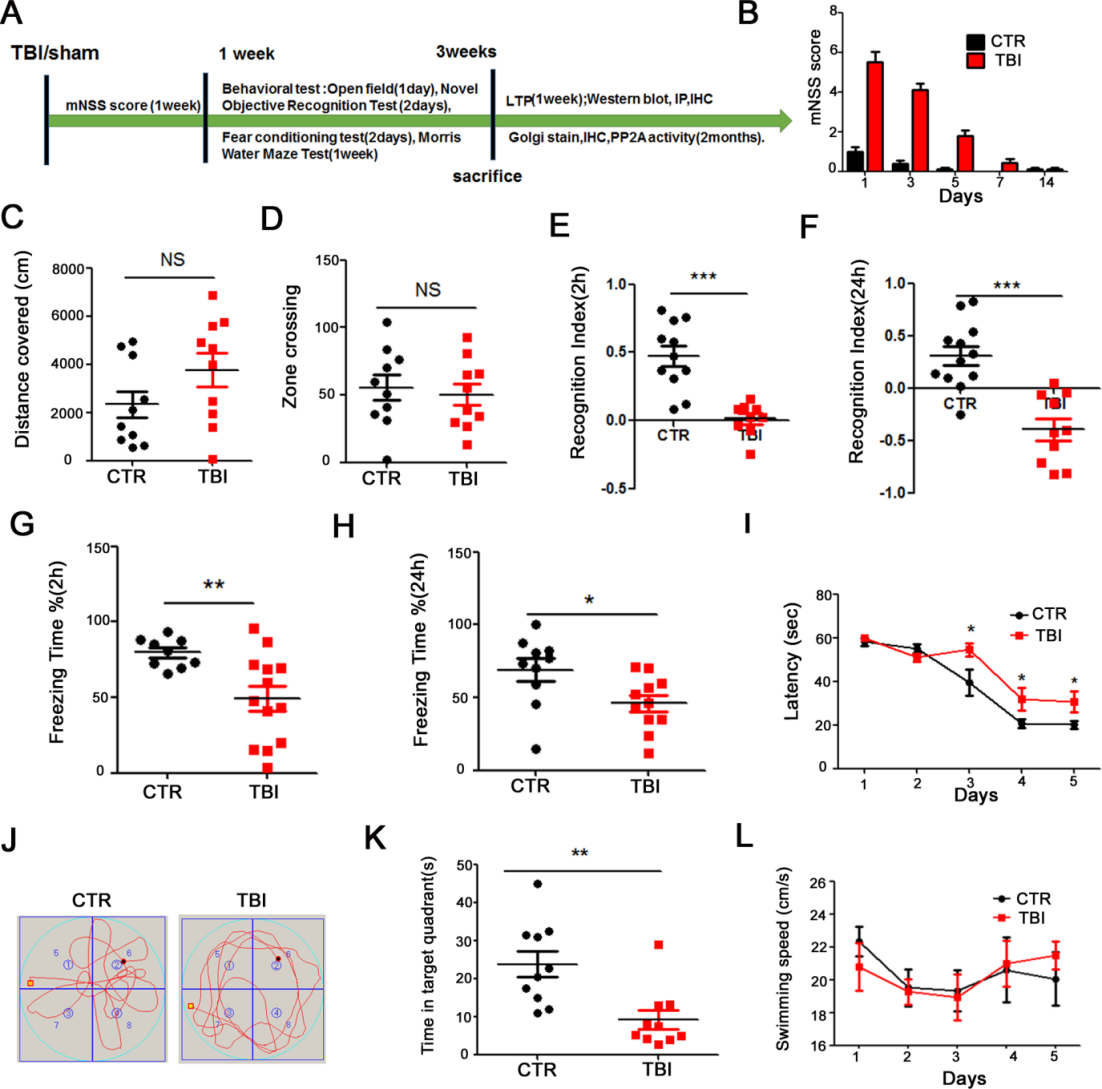
**Traumatic brain injury impaired cognitive functions.** (**A**) Experimental design sketch. (**B**) The mNSS scoring were measured after TBI and in control/sham operation. (**C**, **D**) The open field showed no difference in the total distance covered (**C**) and in the zone crossing (**D**). (**E**, **F**) Novel object recognition test (NOR) showed the measured recognition index of the new object in 2 hours (**E**) and 24 hours (**F**). (**G**, **H**) Fear conditioning test showed the freezing time for 2 hours (**G**) and 24 hours (**H**). (**I**–**L**) In the Morris water maze (MWM) test, the latency to find the hidden platform from day 1-5 (**I**), the spatial memory was tested by removing the platform (**J**) and the time spent in the target quadrant (**K**) and swimming speed (**L**) were measured. n=10/10. *p* value significance is calculated from a one-way ANOVA, data are represented as mean ± SEM. *p < 0.05, **p < 0.01 and ***p < 0.001 *vs* control group.

To investigate the effect of traumatic brain injury (TBI) on cognitive function, twenty-four healthy SD rats were randomly divided into 2 groups (control/sham operation and TBI model). The whole experiment was conducted as depicted in Figure 1A. Following TBI induction, we evaluated the Modified Neurological Severity Score (mNSS). The mNSS scoring results showed that the rats returned to normal motor function one week after TBI (Figure 1B). We then performed a series of behavioral tests. Firstly, open field test was carried and the result showed that there was no significant difference in the total distance covered (Figure 1C), and times in the zone crossing (Figure 1D) between the two groups, indicating that TBI does not affect anxiety or exploratory activity levels. The novelty recognition experiment allows to explore short-term memory capacity, the results from this experiment showed that in the TBI group, the curiosity toward exploring new things were significantly reduced, as the time spent for exploration of the new object in 2 hours (Figure 1E) and 24 hours (Figure 1F) were significantly decreased (*p* value <0.0001). Next, we performed fear conditioning experiment to assess the contextual memory. We found that the TBI rats exhibited significantly lower freezing behavior compared to control in 2 hours (*p* value =0.07) (Figure 1G) and 24 hours (*p* value =0.0236) (Figure 1H). Finally, we tested memory and learning abilities using Morris water maze (MWM), and observed that, compared with the control group, the TBI rats showed significantly increased latency to find the hidden platform (*p* value =0.0434) (Figure 1I). On day 6, the spatial memory was tested by removing the platform. We found a remarkably decreased time spent in the target quadrant in the TBI rats as compared to control group (*p* value =0.0029) (Figure 1J, 1K), while there was no difference in the swimming speed among the two groups (Figure 1L). Together, these findings strongly demonstrate that TBI causes learning and memory impairments in rats.

### Traumatic brain injury led to synaptic dysfunction in the hippocampus of rat

**Figure 2:**
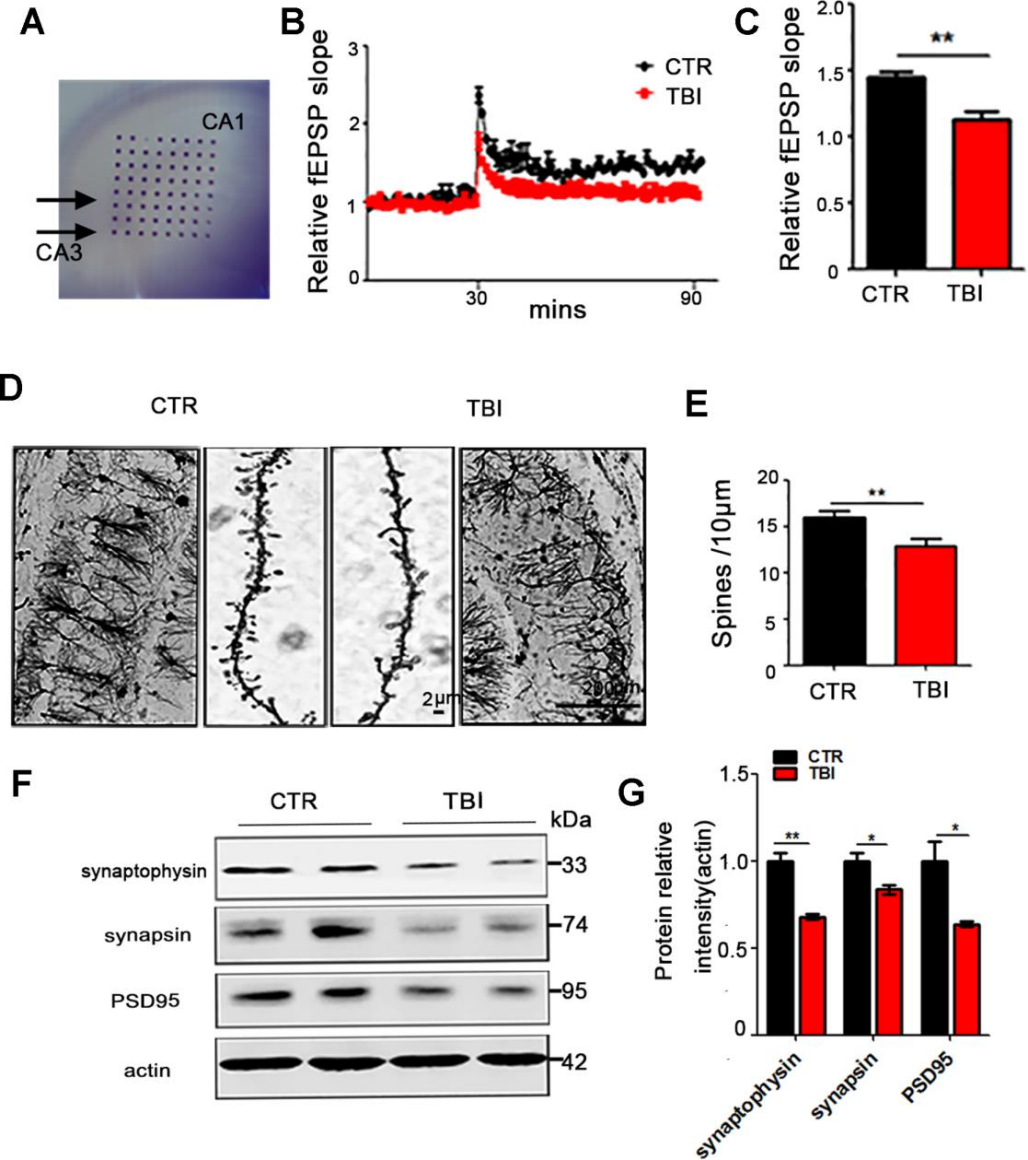
**Traumatic brain injury led to synaptic dysfunction.** (**A**–**C**) Hippocampal CA3-CA1 LTP and its quantification (**A**) were recorded by using the MED64 system. Normalized CA3-CA1 fEPSP mean slope recorded from the CA1 dendritic region in hippocampal slices (**B**, **C**). (**D**, **E**) Representative dendritic spines of neurons from Golgi impregnated hippocampus (**D**) and averaged spine density (**E**) (mean spine number per 10 μm dendrite segment) were measured. Scale bar in left and right lower bar = 200 μm, bar in the middle panel = 2 μm. (**F**, **G**) Brain tissues from hippocampus were homogenized, and synaptic protein levels were detected by immunoblotting. n=3. *p* value significance is calculated from a one-way ANOVA, data are represented as mean ± SEM, *p < 0.05, **p < 0.01 *vs* control group.

The hippocampus (Hip) is an integral part of the limbic system, and is known to play a crucial role in memory and spatial localization which is the reflection of synaptic plasticity. As learning and memory were found to be impaired in TBI rats, accordingly we investigate

whether TBI could induce synaptic dysfunction. To explore how the synaptic transmission in the CA3-CA1 Schaffer collateral (SC) is impaired, hippocampal CA3-CA1 LTP was recorded by using the MED64 system (Figure 2A). We found that TBI rats showed a reduced slope of field excitatory postsynaptic potential (fEPSP) after high-frequency stimulation (HFS) compared with the control group (*p* value = 0.0035) (Figure 2B, 2C). To further investigate the morphological basis of the underlying mechanism, we examined the spine density of hippocampal neurons (Figure 2D and Supplementary Figure 1A). Although no significant difference was found in the total dendritic length (Supplementary Figure 1B), the Golgi staining revealed a significant decrease in the dendritic spine density of the TBI rats (*p* value =0.0029) (Figure 2E) compared with the control. Furthermore, synaptic proteins in the hippocampus of the rat brains were detected by immunoblotting (Figure 2F and Supplementary Figure 1C). A downregulation was observed in the levels of pre-synaptic proteins synapsin (*p* value =0.0347) and synaptophysin (*P* value =0.0029), as well as postsynaptic protein PSD95 (*p* value =0.0437) (Figure 2G and Supplementary Figure 1D). These findings suggest that TBI-induced cognitive impairments might be a reflection of the decreased neural spine density as well as the synaptic dysfunction.

### TBI caused tau hyperphosphorylation and cytoplasmic retention of SET accompanied by AEP activation

**Figure 3:**
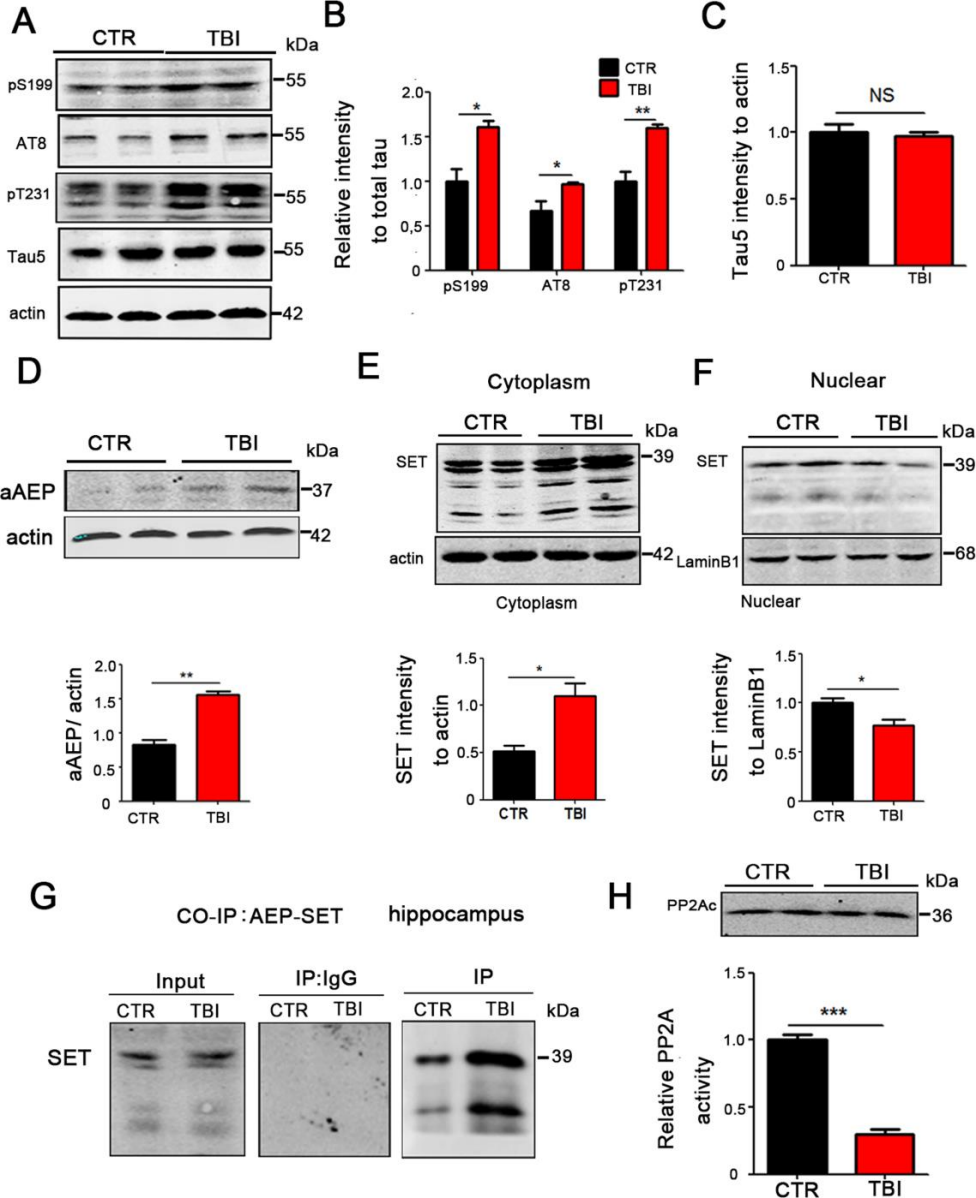
**TBI caused tau hyperphosphorylation and cytoplasmic retention of SET accompanied by AEP activation.** (**A**–**C**) Hippocampal tissues were homogenized, and tau protein levels of pS199, pS202/pT205 (AT8), pT231 were detected by immunoblotting (**A**, **B**). Total tau level was measured (**C**) with actin as loading control. (**D**) aAEP (molecular weight at 36KD) was measured by immunoblotting. (**E**, **F**) We separate cytosolic and nuclear proteins: SET in cytoplasm (**E**) and in nuclei (**F**) were measured. (**G**) The interaction of AEP and SET was evaluated by Co-IP. (**H**) Hippocampal PP2A level and activity were tested. n=3. *p* value significance is calculated from a one-way ANOVA, data are represented as mean ± SEM. *p < 0.05, **p < 0.01 and ***p < 0.001 *vs* control group.

TBI is a high-risk factor for Alzheimer's disease (AD), in which tau is hyperphosphorylated and leads to neuronal impairment and dementia. To explore whether TBI can result in excessive phosphorylation of tau, we performed Western blotting and the result showed a significant increase in the levels of phosphorylated tau at pS199 (*p* value =0.0178), pS202/pT205 (AT8) (*p* value =0.0201), pT231 (*p* value =0.0064) in the TBI model group (Figure 3A, 3B), however there was no significant difference in total tau levels as compared to the control group (Figure 3C). To explore how TBI causes tau hyperphosphorylation, we focused our attention on AEP activity, as this enzyme was reported to be activated in TBI and at the same time involved in Alzheimer’s disease. Western blotting show that the 36KD molecular weight aAEP (active form of AEP [18]) was significantly higher in TBI rats compared with the control (*p* value =0.0012) (Figure 3D). SET also called the inhibitor 2 of PP2A, which is the major tau phosphatase, is mostly found in the nucleus and is activated upon translocation in the cytoplasm. SET has also been reported to be a substrate of AEP. To investigate the interrelation of these proteins in TBI, we separate cytosolic and nuclear proteins and performed Western blotting. The results showed that in the TBI model group SET immunoreactivity in the cytoplasmic fraction was significantly higher compared with control group (*p* value =0.0162) (Figure 3E), however, it was significantly reduced in the nuclear fraction (*p* value =0.0448) (Figure 3F). We also explore the interaction of AEP and SET by co-immunoprecipitation and found a significantly increased interaction of these proteins following TBI (Figure 3G). Finally, we tested hippocampal PP2A activity, and in line with the increased cytoplasmic SET, the TBI rats showed a significantly reduced activity of PP2A compared with control group, but no significant difference in the protein level of PP2Ac (Figure 3H). Together, these data imply that TBI induces AEP activation, and the activated AEP (aAEP) then trapped SET in the cytoplasm, which in turn may explain the increased tau hyperphosphorylation by inhibiting PP2A.

### AEP inhibitor blocked OGD-induced cytoplasmic retention of SET, thereby reducing tau phosphorylation and alleviating the loss of synaptic proteins in hippocampal neurons

**Figure 4:**
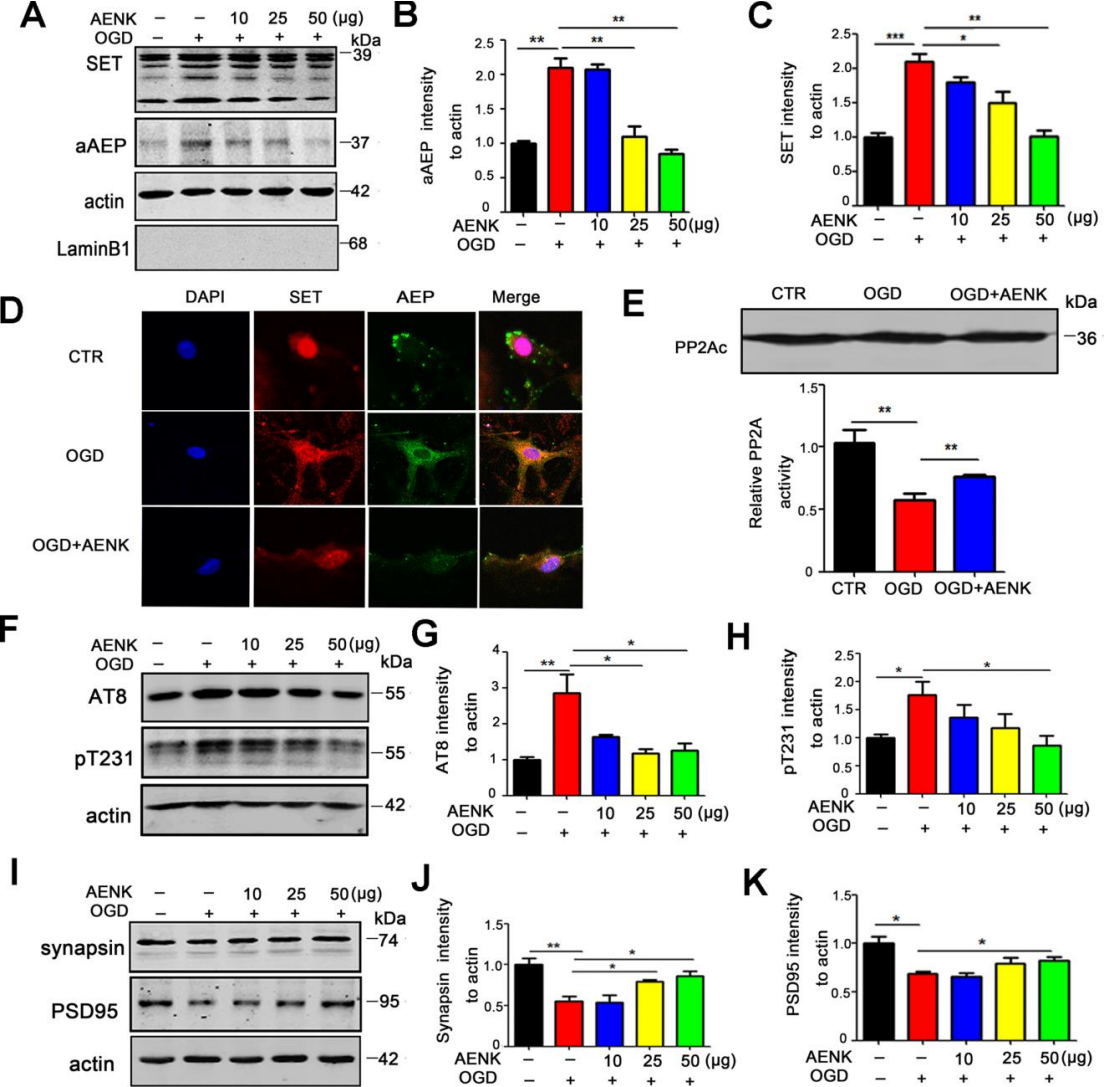
**AENK blocked SET cytoplasmic retention induced by Oxygen-glucose deprivation (OGD), thereby reducing tau phosphorylation and alleviating the loss of synaptic proteins in hippocampal neurons.** (**A**–**C**) Oxygen-glucose deprivation was induced for 12 hours to mimic brain acidification environment. Western blotting (**A**) show that aAEP (molecular weight at 36KD) level was significantly increased in OGD group (**B**) and SET is retained in the cytoplasm(**C**). (**D**) Rat primary hippocampal neurons (DIV7) were incubated with AEP inhibitor/AENK 25 μg for 12 hours following OGD and immunofluorescence staining of SET (red) and AEP (green) were evaluated. (**E**) Primary hippocampal neurons PP2A level and activity were tested. (**F**–**H**) Western blotting (**F**) showed that in the OGD+AENK group the tau levels of AT8 (**G**), pT231 (**H**) were significantly reduced compared with the OGD group. (**I**–**K**) Synaptic proteins were measured (**I**): pre-synaptic proteins synapsin (**J**) and postsynaptic proteins PSD95 (**K**). n=3. *p* value significance is calculated from a one-way ANOVA or two-way ANOVA, data represent mean ± SEM. *p < 0.05, **p < 0.01 and ***p < 0.001 *vs* OGD group.

We found that SET is retained in the cytoplasm leading to tau hyperphosphorylation in TBI, and speculated that AEP could be in part responsible for this. To explore this hypothesis, rat primary hippocampal neurons were cultured for 7 days, and oxygen-glucose deprivation (OGD) was induced for 12 hours to mimic brain acidification environment like TBI. Western blotting from neuronal cell fractions showed that SET was retained in the cytoplasm and aAEP was significantly higher in OGD group (Figure 4A–4C). At the same time with the OGD, the cells were treated with 10μg, 25 μg, and 50 μg doses of AEP inhibitor (AENK). Expectedly, we found that AEP inhibitor resulted in a significant decrease in the 36KD molecular weight aAEP (*p* value =0.0012 at AENK dose 50 μg) (Figure 4A, 4B) with a concomitant blockage or decrease of the cytoplasmic SET retention in OGD+AENK group (*p* value =0.0013 at AENK dose 50 μg) (Figure 4A, 4C). To further corroborate these findings, rat primary hippocampal neurons (DIV7) were incubated with AEP inhibitor (25 μg) for 12 hours under OGD condition and were examined by immunofluorescence staining (Figure 4D). We observed that AEP inhibitor blocked the cytoplasmic retention of SET in OGD+AENK group compared with OGD group (Figure 4D and Supplementary Figure 2, *p* value =0.0037), further supporting that AEP activation induces the cytoplasmic retention of SET. Besides, we tested rat primary hippocampal neurons PP2A activity, and found out that even though the protein level of PP2Ac was not affected, the OGD+AENK group exhibit a significantly increased PP2A activity compared with the OGD group (*p* value =0.0091) (Figure 4E). To explore whether AEP inhibitor affect tau hyperphosphorylation following OGD, the phosphorylation level of tau was evaluated by Western blotting (Figure 4F), and the result revealed that compared with the OGD group, the OGD+AENK group showed a significantly reduced level of phosphorylated tau using pS202/pT205 (AT8) (*p* value =0.045 at AENK dose 50 μg), pT231 (*p* value =0.0348 at AENK dose 50 μg) antibodies (Figure 4G, 4H). Interestingly, an upregulation was observed in the levels of pre-synaptic proteins synapsin (*p* value =0.0252 at AENK dose 50 μg) (Figure 4I, 4J), as well as postsynaptic proteins PSD95 (*p* value =0.0411 at AENK dose 50 μg) (Figure 4I, 4K). These findings suggest that AEP inhibitor blocked the OGD-induced cytoplasmic SET retention, thereby reducing tau hyperphosphorylation and recovering synaptic function in neurons.

### AEP inhibitor alleviated TBI-induced memory dysfunction in rats

**Figure 5:**
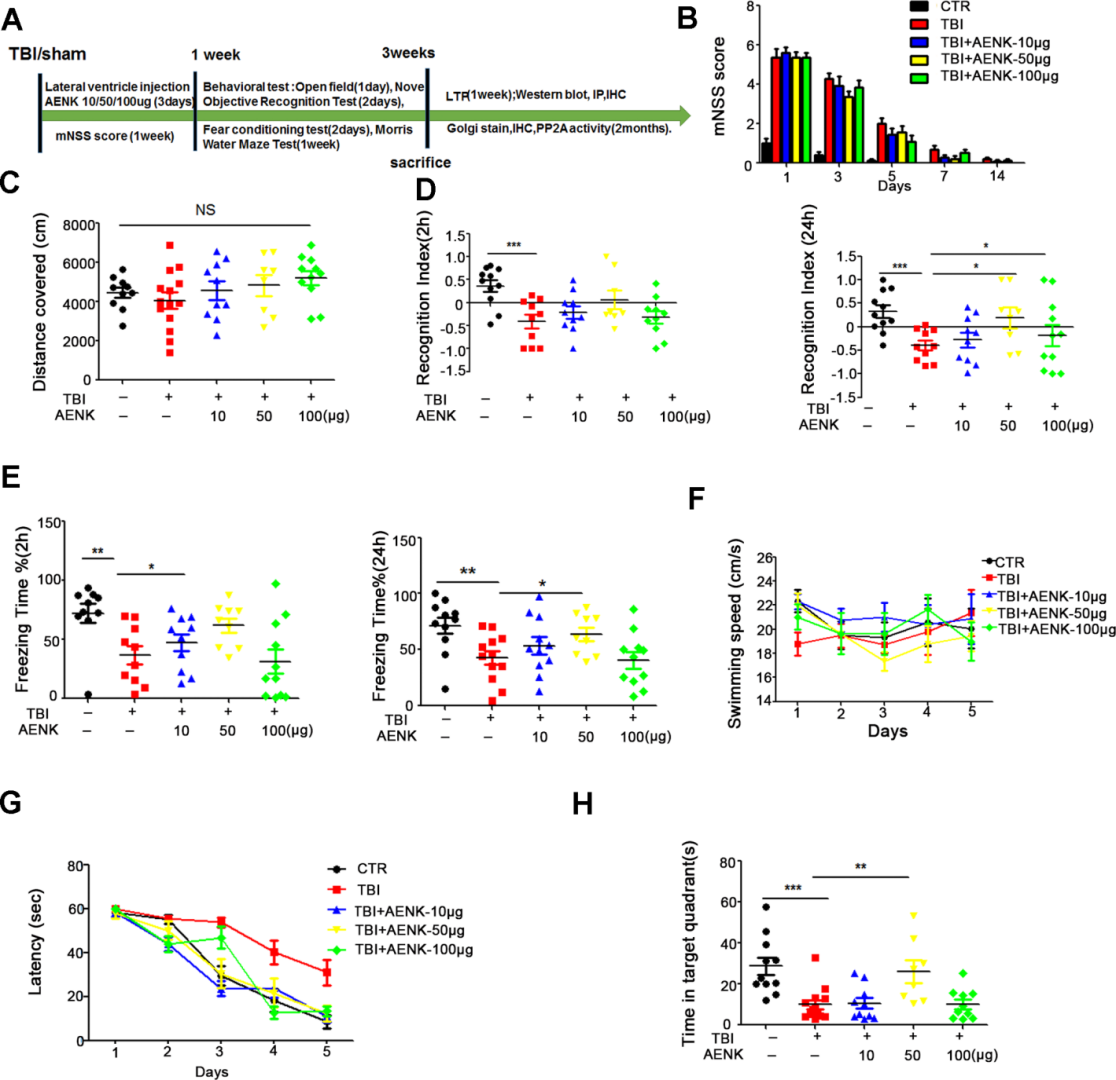
**AEP inhibitor alleviated TBI-induced memory dysfunction.** (**A**) Experimental design sketch. (**B**) The mNSS scoring were measured in all groups following mock and TBI surgeries. (**C**) The open field showed no difference in the total distance covered. (**D**) Novel object recognition test (NOR) showed the measured recognition index of the new object in 2 hours and 24 hours. (**E**) Fear conditioning test showed the freezing time for 2 hours and 24 hours. (**F**–**H**) Morris water maze (MWM) test: swimming speed was measured (**F**), the latency to find the hidden platform from day 1-5 (**G**), and the spatial memory was tested by removing the platform and measuring the time spent in the target quadrant (**H**).n=10-13. *p* value significance is calculated from a one-way ANOVA or two-way ANOVA, all data represent mean ± SEM. *p < 0.05, **p < 0.01 and ***p < 0.001 vs TBI group.

It was shown earlier in this study that TBI induces memory impairments, and activation of AEP was suspected to play a role. To further investigate on that, fifty healthy SD rats were randomly divided into 5 groups of 10-13 animals each, and treated as shown in (Figure 5A). Mock surgery rats served as sham operation control with dimethyl sulfoxide (DMSO) injection into the lateral ventricle, while the remaining are TBI surgery groups and were further divided into four groups as TBI model, TBI +10 μg, +50 μg or +100 μg lateral ventricular injection of AEP inhibitor. We evaluated the mNSS, and the scoring results showed that the rats returned to normal motor function a week after surgery (Figure 5B). The same series of behavioral tests were carried out, and the open field test result showed that there was no significant difference in the total distance covered (Figure 5C), suggesting no difference in locomotor functions among the groups. The results of novelty recognition experiment however showed that in the TBI+AENK groups, the curiosity toward exploring new things was significantly increased, as the time spent for exploration of new object in 24 hours was significantly higher (*p* value =0.0211 at AENK dose 50μg), although no difference change was observed in 2 hours evaluation (Figure 5D). Next, we performed fear conditioning experiment to assess the contextual memory. We found that the TBI+AENK groups exhibited significantly higher freezing behavior (Figure 5E), compared to control in 2 hours evaluation (*p* value =0.336 at AENK dose 10 μg) and in 24 hours (*p* value =0.031 at AENK dose 50 μg). Finally, we tested memory and learning abilities using MWM and found no significant changes in swimming speed among all groups (Figure 5F), but observed a significantly reduced latency to find the hidden platform in the TBI+AENK rats (Figure 5G). On day 6, the spatial memory was tested by removing the platform, and a remarkable increase in the time spent in the target quadrant (Figure 5H) was observed in the TBI+AENK rats compared with the TBI group (*p* value =0.00652 at AENK dose 50 μg). Together, these data demonstrate that AEP inhibitor could restore learning and memory impairments in TBI rats, further suggesting the implication of AEP in AD-like pathology following TBI.

### Blockage of AEP abrogated the cytoplasmic retention of SET and attenuated tau pathology in TBI rats

**Figure 6:**
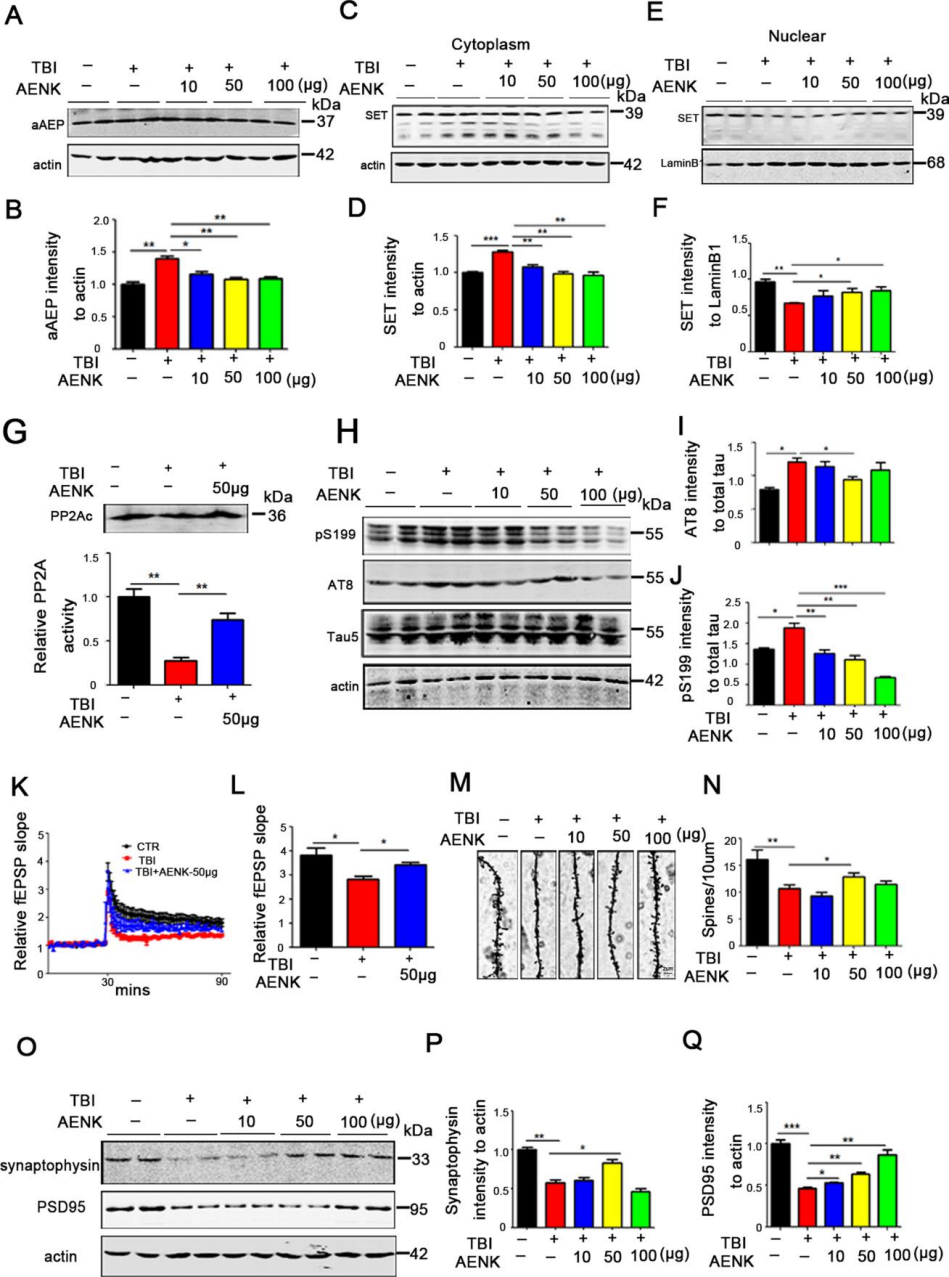
**Blockage of AEP abrogated the cytoplasmic retention of SET and attenuated tau pathology in TBI rats.** (**A**, **B**) aAEP (molecular weight at 36KD) was significantly inhibited in TBI+AENK group, compared with the TBI group as evaluated by Western blotting. (**C**, **D**) The level of SET was significantly reduced in cytosolic fraction, but was increased in the nucleus (**E**, **F**) in TBI+AENK group compared with TBI group. (**G**) Brain hippocampal neurons PP2A level and activity were tested. (**H**–**J**) Western blotting (**H**) show significantly reduced levels of AT8 (**I**), pS199 (**J**) in the TBI+AENK group compared with the TBI group. (**K**, **L**) Normalized CA3-CA1 fEPSP mean slope recorded from the CA1 dendritic region in hippocampal slices. (**M**, **N**) Representative dendritic spines of neurons from Golgi impregnated hippocampus (**M**). Average spine density (**N**) (mean spine number per 10 μm dendrite segment) were measured. Scale bar = 2 μm. (**O**–**Q**) Hippocampal tissues were homogenized, and synaptic protein levels were detected by immunoblotting (**O**). Pre-synaptic proteins synaptophysin (**P**) and postsynaptic proteins PSD95 (**Q**). n=3. *p* value significance is calculated from a one-way ANOVA or two-way ANOVA, all data represent mean ± SEM. *p < 0.05, **p < 0.01 and ***p < 0.001 vs TBI group.

We have proposed that TBI leads to cytoplasmic retention of SET and tau hyperphosphorylation by upregulating AEP activity. Interestingly, AEP inhibitor restored learning and memory impairments in TBI rats. To explore the restorative effect of AEP inhibitor on learning and memory in TBI+AENK rats, we analyzed the immunoreactivity of the activated AEP (36KD aAEP) by Western blotting and found out that it was significantly reduced in TBI+AENK groups compared with the TBI one (*p* value =0.0026 at AENK dose 50 μg) (Figure 6A, 6B). We also separate cytosolic and nuclear fractions to explore the effect of AEP inhibitor on the cytosolic retention of SET by Western blotting. The result showed that in the cytosolic fraction from TBI+AENK groups, the protein level of SET was significantly reduced compared with TBI group (*p* value =0.0023 at AENK dose 50 μg) (Figure 6C, 6D). However, the nuclear protein level of SET was increased in TBI+50/100 μg AENK groups (*p* value =0.0472 at AENK dose 50 μg) (Figure 6E, 6F), together indicating a blockage of the AEP-induced cytoplasmic retention with a nuclear relocation of SET by the AEP inhibitor. Next, we measured hippocampal PP2A activity of TBI+50 μg AENK group, which revealed a significantly increased PP2A activity in the TBI+AENK group compared with TBI group (at AENK dose 50 μg) with no effect on the protein level of PP2Ac (*p* value =0.0042 at AENK dose 50 μg) (Figure 6G). As a major tau phosphatase PP2A decreases the level of tau phosphorylation. PP2A activity was shown to be increased following AEP inhibitor administration. As a consequence of that, Western blotting (Figure 6H) show that the TBI+AENK group have significantly reduced level of tau phosphorylation at pS202/pT205 AT8 (*p* value =0.0256 at AENK dose 50 μg) (Figure 6I), pS199 (*p* value =0.0079 at AENK dose 50 μg) (Figure 6J) compared with the TBI group. These findings further imply that AEP-induced SET cytoplasmic retention leads to PP2A inhibition and tau hyperphosphorylation in TBI brain.

Since synaptic alterations were observed in TBI rats (Figure 2G), we wonder whether AEP could alter synaptic plasticity, and that AEP inhibitor might block or recover this effect. To answer this curiosity, hippocampal CA3-CA1 LTP was recorded by using the MED64 system. We found that TBI induced a decrease in the slope of field excitatory postsynaptic potential (fEPSP) after high-frequency stimulation (HFS) compared with the mock surgery control group, while this effect was significantly recovered in the TBI+AENK group (*p* value =0.01 at AENK dose 50 μg) (Figure 6K, 6L). To understand to anatomical basis of these changes, we further examined the spine density of hippocampal neurons (Figure 6M). The result revealed a significant increase in the dendritic spine density of the TBI+AENK rats (at AENK dose 50 μg) compared with the TBI group (*p* value =0.0249 at AENK dose 50 μg) (Figure 6N). Furthermore, we examined synaptic proteins in the hippocampus of the rat brains by immunoblotting (Figure 6O), and an upregulation was observed in the levels of pre-synaptic proteins synaptophysin (*p* value =0.0135 at AENK dose 50 μg) (Figure 6P), as well as postsynaptic proteins PSD95 (*p* value =0.0034 at AENK dose 50 μg) (Figure 6Q). These findings suggest that AEP inhibitor blocked or at least decreased the AEP-induced cytosolic retention of SET and at the same time reduced tau hyperphosphorylation, alleviated synaptic damage and improved synaptic plasticity.

## DISCUSSION

TBI has been considered a significant risk factor for the development of AD, which is characterized by cognitive dysfunction, tau hyperphosphorylation and senile plaques. However, the mechanism of how TBI induced AD is still unclear. In the current work, we explore the molecular mechanism whereby TBI exerts its deleterious effect on cognitive functions and found out that TBI induces tau hyperphosphorylation and synaptic dysfunction. Noticeably, the pathway via which TBI leads to tau pathology is dependent on I_2_^PP2A^ (inhibitor 2 of PP2A, also called SET) cytoplasmic retention and the activation of asparaginyl endopeptidase (AEP), a lysosomal cysteine protease, which is activated under acidic milieu and is associated with the process of Alzheimer's disease (AD) [26]. Therefore, in order to explore AEP activation and tau pathology, oxygen-glucose deprivation (OGD) was induced in rat primary hippocampal neurons to mimic brain acidification environment like in TBI. We found that OGD leads to AEP activation, SET translocation from neuronal nucleus to cytoplasm, PP2A inhibition, tau hyperphosphorylation and a decrease in synaptic proteins in neurons. Interestingly, blockage of AEP activation with AEP inhibitor obviously decreased OGD-induced cytoplasmic SET retention, subsequently reducing tau hyperphosphorylation and recovering synaptic function in primary neurons. Moreover, AEP inhibitor restores SET back to nucleus, mitigates tau pathologies and improves synaptic function, consequently rescuing TBI-induced cognitive impairment in rats. Together, our data strongly support the notion that blockage of AEP attenuates TBI-induced tau hyperphosphorylation and cognitive impairments in rats.

Repetitive mild TBI and severe TBI have been confirmed as a risk factor for dementia and Alzheimer's disease (AD) [26, 39, 42]. Given that dementia may result from numerous underlying pathologies, advanced knowledge of how TBI acts as a risk factor of neuropathological alteration requires to be further addressed. Walker and Tesco reported that, as a direct insult of TBI, Aβ pathology might exacerbate secondary injury mechanisms thereby establishing a neurotoxic cascade that leads to chronic neurodegeneration [43, 44]. Resolvin D1 (RvD1), an important endogenous specialized pro-resolving mediator, has been also reported to control neuroinflammation and protect from astrocytic mitochondria dysfunction induced by TBI, suggesting that RvD1 may have a potent therapeutic value in ameliorating cognitive impairment following TBI [45, 46]. In the current study, we further investigated the effect of moderate-to-severe TBI-induced tau pathology on cognitive impairments and its related mechanisms. We found that TBI cause learning and memory impairments in rats, which might be associated with the decreased neural spine density as well as the synaptic dysfunction. Moreover, TBI induces AEP activation, and then the activated AEP (aAEP) trapped SET in the cytoplasm, which in turn results in tau pathology. These findings are in alignment with recent observations that repetitive mild TBI results in tau hyperphosphorylation and activation of AEP [26]. As a lysosomal cysteine protease [20], AEP is highly activated in AD patients and cleaves SET, and subsequently enhances tau hyperphosphorylation via inhibiting PP2A by truncated SET [9, 21]. To further confirm that active AEP traps SET in the cytoplasm and induces tau pathology, TBI-like acidic environment was mimicked by OGD. We found that blockage of AEP impeded both its interaction with SET and the OGD-induced cytoplasmic SET retention, thereby reducing tau hyperphosphorylation and recovering synaptic function in neurons. Hence, AEP interaction with SET might be responsible of the observed tau pathology. Meanwhile, it has been shown that AEP-induced cytoplasmic retention of SET induces full length SET truncation into its N and C terminal fragments, both of which inhibit PP2A activity and lead to tau hyperphosphorylation [9, 21].

Previous studies show that cytoplasmic SET accumulation plays a role in tau hyperphosphorylation and cognitive defects *in vivo* [47]. In the present study, TBI rats display cytoplasmic SET and cognitive impairments accompanied with AEP activation and an increase in AEP binding to SET, suggesting that AEP traps SET and mediates cognitive dysfunction via tau pathology. Interestingly, AEP inhibitor/AENK decreased the AEP interaction with SET and the cytosolic SET retention, thereby reducing tau phosphorylation, alleviating synaptic damage, finally restoring learning and memory impairments in TBI rats.

In summary, our findings support that TBI induces AEP activation and increases SET retention in the cytoplasm, where it inhibits PP2A, leading to tau hyperphosphorylation and cognitive impairments. Given the deteriorative effect of hyperactivation of AEP in TBI exerts numerous pathological events, inhibition of AEP by its inhibitors might provide pharmacological intervention for treating TBI-induced neurological disease.

## MATERIALS AND METHODS

### Reagents

AENK (AEP inhibitor) was purchased from Dalian Meilun (Number of products: MB2484). PP2A activity kit was purchased from Promega (V2460). Antibodies employed in this study are listed in Table 1.

**Table 1:** Antibodies employed in this study.

**Antibodies**	**Specific**	**Type**	**Dilution**	**Sources**
PSD95	Total PSD95	pAb	1/1,000	Cell Signaling
AEP	Legumain/Asparaginyl Endopeptidase	pAb	1/1000	R&D
SET	I_2_^PP2A^	pAb	1:1000 for WB 1:50 for IP	Santa Cruze
PP2Ac	PP2AC	mAb	1/1000	Millipore
Tau-5	Total-tau	mAb	1:1000 for WB	Lab Vision
pS199	Phosphorylated Tau at Ser199	pAb	1:1000 for WB	Invitrogen
pT231	Phosphorylated Tau at Thr 231	pAb	1:1000 for WB	SAB
AT8	Phosphorylated Tau at Ser202/Thr205	mAb	1:1000 for WB	Thermo
NR1	C-terminus (amino acids 909-938)	mAb	1/500	Millipore
NR2B	C-terminus (amino acids 984-1104)	pAb	1/500	Millipore
NR2A	C terminal last 200 amino acids	pAb	1/1,000	Abcam
GluA1	GluA1 protein	pAb	1/500	Millipore
GluA2	GluA2 (amino acids 175–430)	pAb	1/500	Millipore
Synapsin1	Synapsin 1	pAb	1/1,000	Millipore
Synaptophysin	Synaptophysin	mAb	1/1,000	Sigma
β-actin	β-actin	mAb	1/1,000	Abcam
LaminB1	LaminB1	pAb	1/1,000	Abcam

I_2_^PP2A^, inhibitor 2 of PP2A; mAb, monoclonal antibody; pAb, polyclonal antibody; WB, Western blot; IP, immunoprecipitation.

### Animals

Male Sprague-Dawley rats (2 months old, 250 ± 20 g) were supplied by the Experimental Animal Center of Tongji Medical College, Huazhong University of Science and Technology. Rats were kept under standard laboratory conditions: 12-hours light and 12-hours dark with water and food ad libitum. Rats were randomly divided into groups and treated as stated in different part of the study.

### TBI model

After 2 weeks of adaptive feeding, two and half months old rats were subjected to operation under anesthetization (50 mg/kg sodium pentobarbital; Boster Biotech, Wuhan, China; intraperitoneally). The rats were placed on a heating pad to maintain body temperature at 37 °C to prevent hypothermia. The head of each rat was fixed in a stereotaxic frame. Briefly, a 3 cm midline incision was made over the skull, and a 5-mm craniotomy was drilled through the skull 2 mm caudal to the left coronal suture and 2 mm from the mid-line without disturbing the dura. TBI was induced using a weight drop hitting device (ZH-ZYQ, Electronic Technology Development Co., Xuzhou, China) with a 4.5-mm-diameter cylinder bar weighing 40 g from a height of 20 cm, when no hitting rats were served as sham control. Bone wax was used to seal the hole, and the scalp was sutured [48]. All the rats were awake after the operation.

### Assessment of neurological injury

Modified neurological severity scores (mNSS) were used to evaluate the motor (muscle state, abnormal movement), sensory (visual, tactile, and proprioceptive), and reflex systems of rats. The mNSS test is graded on a scale of 0-18, where a total score of 18 points indicates severe neurological deficits and a score of 0 indicates normal performance; 13-18 points indicates severe injury, 7-12 indicates mean to moderate injury, and 1-6 indicates mild injury [49]. Evaluation was performed 1, 3, and 7 days after TBI by investigators who were blinded to the experiments.

### Lateral ventricle stereotactic surgery

The rats were anesthetized with isoflurane and placed in a stereotaxic apparatus. After being sterilized with iodophors and 75% (vol/vol) alcohol, the scalp was incised along the midline between the ears. Holes were drilled in the hibateral skull stereotaxically at posterior 1.2 mm, lateral 2.6 mm, and depth 4.0 mm relative to bregma. Using a microinjection system (World Precision Instruments), AEP inhibitor/AENK was injected immediately after Traumatic Brain injury into contralateral Lateral ventricle (5 μL) at a rate of 0.25 μL/min, the needle was kept in place for 10 min before withdrawal, the skin was sutured. Sham mice were subject to anesthesia for the same amount of time for TBI and Lateral ventricle stereotactic surgery.

### Behavior Test

### Open field

The open field is used to assess anxiety and exploration activities. The test equipment is a typical open field (100 x 100 cm 2 PVC square arena with 70 cm high walls). Rats were individually trained for a single 5 minutes period. Anxiety is studied by analyzing the percentage of time spent in the middle of the arena. To assess the exploration activity, the total distance covered by the animal in the box was tracked and measured.

### Novel Objective Recognition Test

The rats were placed in the arena (100 cm x 100 cm x 70 cm container) for 5 min. The day after, the rats reentered the arena from the same starting point, they were granted 5 min to familiarize themselves with object A and object B. Exactly 2 hours after the familiarization period, object B (familiar object) was replaced with object C (new object), and the rat were granted 5 min to explore both objects. After 24 hours, object C (now familiar object) was replaced with object D (now new object), and the rats were granted 5 min to explore both objects. The behavior was recorded by a video camera positioned above the arena. The recognition index was calculated as TA/(TA + TB), TB/(TA + TB), TC/(TA + TC), and TD/(TA + TD). The discrimination index was calculated as (TC - TA)/(TA + TC), (TD -TA)/(TA + TD). TA, TB, TC, and TD were, respectively, the times mice explored the objects A, B, C, and D.

### Fear conditioning test

The test included two periods. The first period involved training: rats were placed in the chamber and after 3 min a sound stimulus was administered for 10 sec, then short-term current stimulation (0.8 mA, 3 s) immediately followed. The current stimulation cycle was repeated three times. The second period is the test: this period take place either 2 hours or 24 hours following the training and involved only sound stimulation, with no current administered. The freezing times, as the rats await the short-term current stimulus, were then recorded.

### Morris Water Maze Test

Spatial memory was detected by the Morris water maze (MWM) test. The temperature of the water was monitored each day to ensure rats were tested in water between 22 °C and 24 °C. For spatial learning, the rats were trained in the water maze to find a hidden platform for 5 days, four trials per day from 8:00 am to 14:00 pm. In each trial, the rats started from one of four quadrants facing the wall of the pool and ended when the animal climbed on the platform. If the rats failed to find the platform within 60 s, they were guided to the platform and allowed to stay on the platform for 20 s uniformly. The swimming path and the time used to find the platform (latency) were recorded by a tracking video camera. Spatial memory was tested next day after training. The platform was removed and the time spent in target quadrant and swimming speed were recorded for 60 s.

### Golgi Staining

To evaluate the effects of AENK on neural morphology in the neurons of TBI rats, a modified Golgi stain method was used [50]. The rats were anesthetized by isoflurane and perfused intracardially with 400 mL of normal saline, followed with 400 mL of 4% formaldehyde and the Golgi dye solution containing 5% chloral hydrate, 4% formaldehyde, and 5% potassium dichromate. After being perfused, the brains were transferred to a vial containing Golgi dye solution for 14 days in the dark. The brains were serially sectioned into 100-μm-thick slices using a vibrating microtome (Leica, VT1000S, Germany). Images were observed under a light microscope (Nikon, Tokyo, Japan).

### Long-term potentiation (LTP)

LTP was measured using the MED64 multielectrode array (Alpha Med Sciences, Kadoma, Japan) as described previously [50, 51]. Briefly, acute brain slices were transferred to a recording chamber and submerged in artificial CSF (aCSF). Slices were laid down in a chamber with an 8 × 8 microelectrode array (Parker Technology, Beijing, China) in the bottom planar (each 50 × 50 mm in size, with an interpolar distance of 150 μm) and kept submerged in the aCSF. The field excitatory postsynaptic potentials (fEPSPs) in CA1 neurons were recorded by stimlating CA3 neurons. LTP was induced by applying three trains of high-frequency stimulation (HFS; 100 Hz, 1-s duration). The LTP magnitude was quantified as the percentage change in the fEPSP slope (10%−90%) taken during the 60-min interval after LTP induction.

### Assessment of PP2A activity

PP2A activity analysis carried out in line with standard procedures as described previously [52]. PP2A activity in brain hippocampus tissue samples was measured using the phosphatase kit (V2460 Promega) according to the manufacturer’s procedure.

### Primary cultures of hippocampal neurons

Primary cultures of rat hippocampal neurons were prepared from E18 Sprague–Dawley rat embryos as previously reported. Primary cultures of hippocampal neuron were performed as previously described [44, 46]. Briefly, the hippocampus was dissected and gently chopped in Hank's buffered saline solution and then suspended in a 0.25% (v/v) trypsin solution for 15 minutes at 37 °C. Neurons were plated in 6-well and 12-well plates coated with 100 μg/mL poly-D-lysine and supplemented with 2% (v/v) B-27 and 1×GlutaMAX. Half of the media was changed every 3 days. Hippocampal neurons were cultured at 37 °C in a humidified 5% (vol/vol) CO_2_ incubator before treatment. The neurons were cultured for 7 days and then treated. At the end of treatments, cells were collected and lysed in RIPA buffer for further biological detections or fixed with 4% paraformaldehyde for immunofluorescence imaging. All cell culture reagents were purchased from Thermo Fisher Scientific.

In the preliminary experiment, we have tested the concentration gradient of AENK *in vitro.* We first selected 6 concentrations of 1 μg/ml, 10 μg/ml, 25 μg/ml, 50 μg/ml, 100 μg/ml, 200 μg/ml to treat with primary hippocampal neuron cells. We found that at 100 and 200 μg/ml, AENK cause the primary neuron cells shrunk and dead within 24 hours. Therefore, we chose 10 μg/ml, 25 μg/ml and 50 μg/ml of AENK *in vitro* experiments.

### Immunofluorescence

Immunofluorescence analysis were carried out in line with standard procedures as described previously [50, 52]. Hippocampus neurons were fixed in 4% paraformaldehyde for 10 min, washed with PBS, and blocked in 3% BSA and 0.5% Triton X-100 for 30 min, and labeled via overnight incubation with primary-antibodies at 4 °C. The neurons were washed three times in PBS and incubated with the secondary antibodies conjugated to Alexa-Fluor 488/546 for 1 hour at room temperature, and washed in PBS and the nuclei were stained with DAPI (1:1000) for 10mins. The neurons were washed again three times in PBS, and mounted by covering with glass coverslips using mounting solution. Cells were examined using confocal microscopy (LSM710, Zeiss, Germany).

### Western Blotting and Co-immunoprecipitation (Co-IP)

Western blot and Co-immunoprecipitation analysis were carried out in line with standard procedures as described previously [50, 53]. Cell or rats brain tissue samples were lysed with RIPA supplemented with protease inhibitor PMSF and cocktail (catalog number: P8340, from Sigma-Aldrich), and then centrifuged for 10 min at 12,000 g. Supernatants were boiled in SDS loading buffer and protein separated using SDS–PAGE. Proteins were then transferred to nitrocellulose membranes. Western blotting analysis was performed using the indicated antibodies in Table 1.

Nuclear and cytoplasmic protein preparation kit (P1200, Pulilai) was used to isolate the nucleus and cytoplasm fractions from brain hippocampal tissue samples for subsequent experiments according to the manufacturer's procedure.

To analyze protein-protein interactions, we performed Co-IP experiments using hippocampus lysates. Specified antibody and protein G agarose were incubated with the samples overnight at 4°C. The resins were washed three times with PBS. After elution by 2× loading buffer, and boiled at 95°C for 10 min, the bound proteins were analyzed by western blotting.

### Statistical analysis

Data were expressed as mean ± SEM and analyzed using GraphPad statistical software. The one-way ANOVA was used to determine the differences among groups. For the comparison between two groups, the Student’s t test was used. The significance was assessed at *p* < 0.05.

### Ethics approval and consent to participate

All animal experiments were approved by the Animal Care and Use Committee of Huazhong University of Science and Technology, and performed in compliance with the National Institutes of Health Guide for the Care and Use of Laboratory Animals.

### Availability of data and materials

The datasets used and/or analyzed during the present study are available from the corresponding author on reasonable request.

## Supplementary Material

Supplementary Figures
