# Correction for: Blockage of AEP attenuates TBI-induced tau hyper-phosphorylation and cognitive impairments in rats

**DOI:** 10.18632/aging.205989

**Published:** 2024-06-15

**Authors:** Yi Liu, Cuiping Guo, Yi Ding, Xiaobing Long, Wensheng Li, Dan Ke, Qun Wang, Rong Liu, Jian-Zhi Wang, Xiaochuan Wang, Huaqiu Zhang

**Affiliations:** 1 Department of Pathophysiology, Weifang Medical University, Weifang 261053, China; 2 Department of Pathophysiology, School of Basic Medicine, Key Laboratory of Education Ministry of China for Neurological Disorders, Tongji Medical College, Huazhong University of Science and Technology, Wuhan 430030, China; 3 Department of Neurosurgery, Tongji Hospital, Tongji Medical College, Huazhong, University of Science and Technology, Wuhan 430030, China; 4 Co-Innovation Center of Neuroregeneration, Nantong University, Nantong 226001, China

**Keywords:** traumatic brain injury (TBI), asparaginyl endopeptidase (AEP), AEP inhibitor/AENK, tau pathology, cognitive impairment

**This article has been corrected:** The authors recently found that they used the wrong images in Supplementary Figure 1C and Figure 3A. The western blot image “Glur1” in Supplementary Figure 1C is a duplication of the “PSD95” western blot image in Figure 4I. This inadvertent mistake was corrected by replacement of the incorrect image with the “Glur1” western blot image from the initial set of experiments. The authors provided uncropped images of the original blots. They also found that the western blot image for "actin" in Figure 3A came from the folder for Figure 2 and replaced it with the correct image from the original western blot data. These corrections do not change the content of the publication and do not affect the conclusion drawn from this research. The authors would like to apologize for any inconvenience caused.

The corrected version of **Figures 3**** and ****Supplementary Figure 1** are provided below.

**Figure 3:**
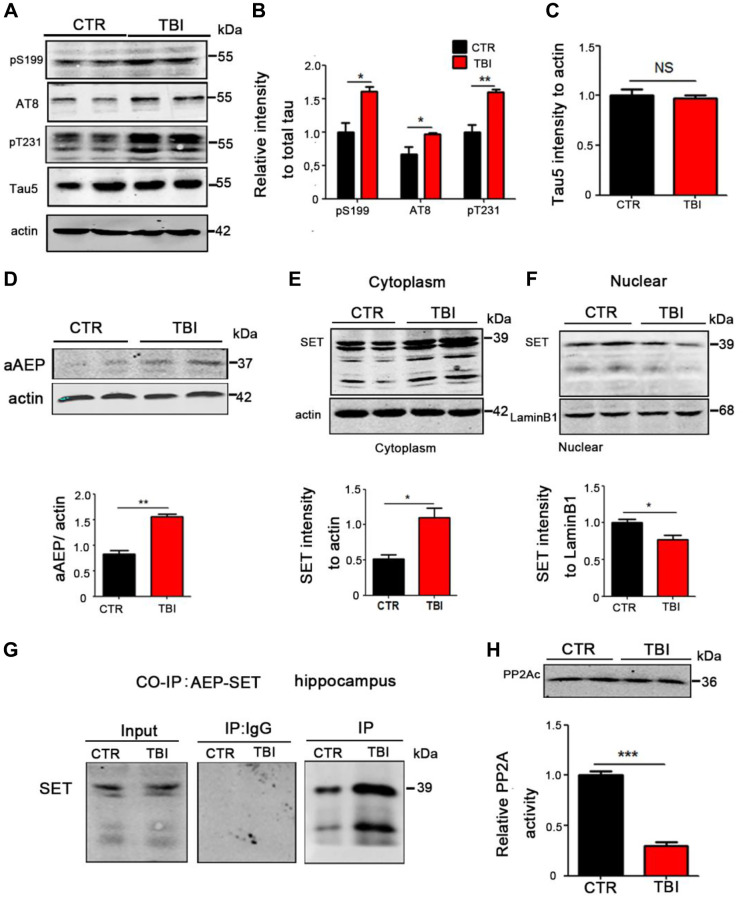
**TBI caused tau hyperphosphorylation and cytoplasmic retention of SET accompanied by AEP activation.** (**A**–**C**) Hippocampal tissues were homogenized, and tau protein levels of pS199, pS202/pT205 (AT8), pT231 were detected by immunoblotting (**A**, **B**). Total tau level was measured (**C**) with actin as loading control. (**D**) aAEP (molecular weight at 36KD) was measured by immunoblotting. (**E**, **F**) We separate cytosolic and nuclear proteins: SET in cytoplasm (**E**) and in nuclei (**F**) were measured. (**G**) The interaction of AEP and SET was evaluated by Co-IP. (**H**) Hippocampal PP2A level and activity were tested. *n* = 3. *P*-value significance is calculated from a one-way ANOVA, data are represented as mean ± SEM. ^*^p < 0.05, ^**^p < 0.01 and ^***^p < 0.001 vs. control group.

**Supplementary Figure 1:**
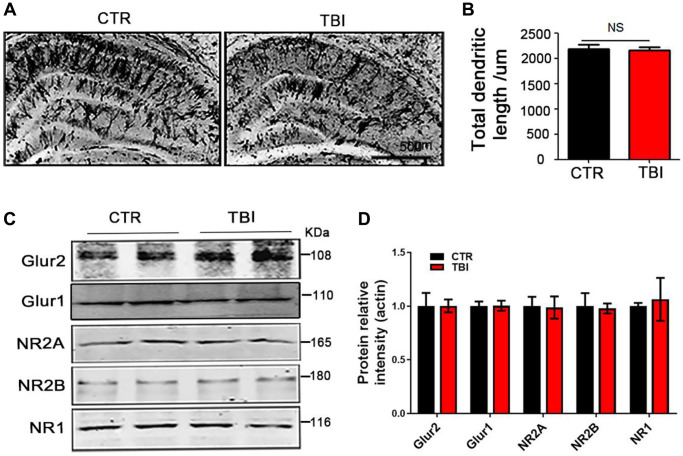
**Traumatic brain injury led to synaptic dysfunction.** (**A**) Representative dendritic spines of neurons from Golgi impregnated hippocampus. Scale bar = 500 μm. (**B**) Total dendritic length. (**C**, **D**) Brain tissues from hippocampus were homogenized, and synaptic protein levels were detected by immunoblotting, *n* = 3. *P*-value significance is calculated from a one-way ANOVA, all data represent mean ± SEM.

