# Supplementary material for: Blockage of AEP attenuates TBI-induced tau hyperphosphorylation and cognitive impairments in rats
Source: Aging (Albany NY). 2020 Oct 10;12(19):19421–39. doi: 10.18632/aging.103841 (PMC7732271; doi:10.18632/aging.103841)
Supplement: Supplementary Figures [file aging-12-103841-s001..pdf]

SUPPLEMENTARY FIGURES

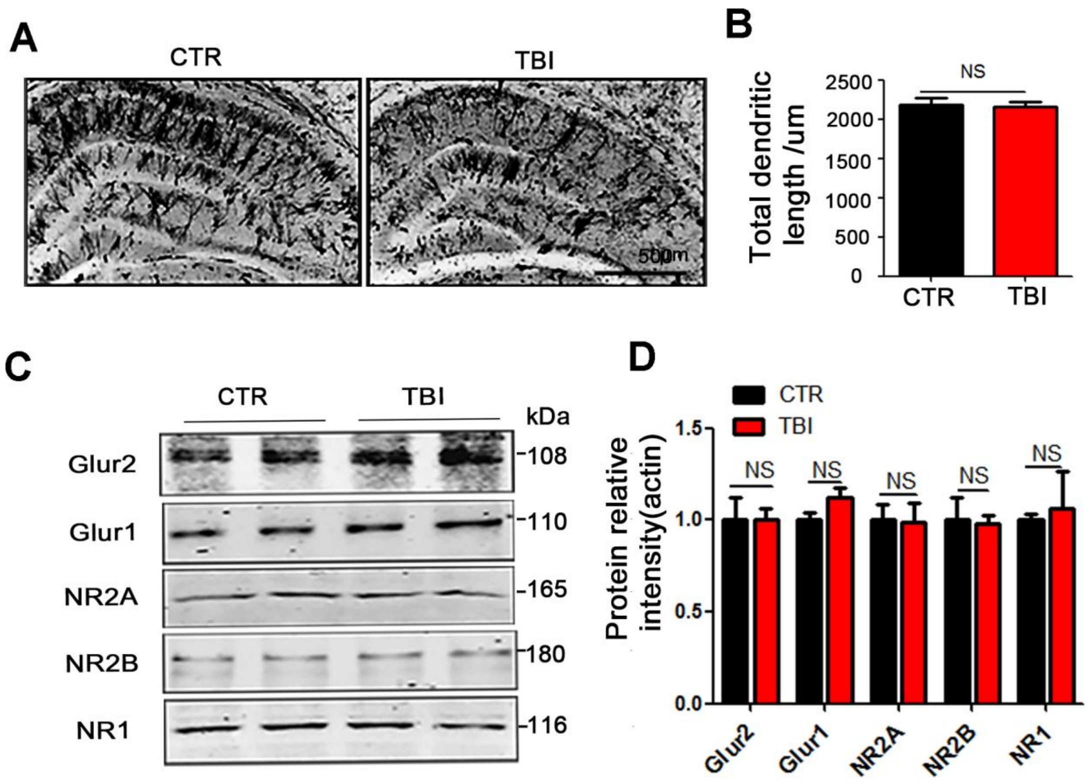

Supplementary Figure 1. Traumatic brain injury led to synaptic dysfunction. (**A**) Representative dendritic spines of neurons from Golgi impregnated hippocampus. Scale bar = 500 µm. (**B**)Total dendritic length. (**C**, **D**) Brain tissues from hippocampus were homogenized, and synaptic protein levels were detected by immunoblotting, n=3. *p* value significance is calculated from a one-way ANOVA, all data represent mean ± SEM.

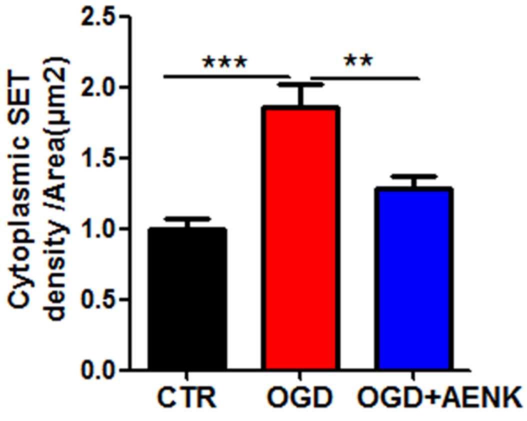

Supplementary Figure 2. OGD caused SET cytoplasmic detention. Quantitative analysis of the immunofluorescence staining showed the cytoplasmic SET density significantly increased by OGD processing. n=30 cells. *p* value significance is calculated from a two-way ANOVA, all data represent mean ± SEM, ***p* < 0.01, ****p* < 0.001 *vs* OGD group.
